# Cx43 upregulation in HUVECs under stretch via TGF-β1 and cytoskeletal network

**DOI:** 10.1515/med-2022-0432

**Published:** 2022-03-09

**Authors:** Yumeng Shi, Xinbo Li, Jin Yang

**Affiliations:** Department of Ophthalmology and Visual Science, Eye Ear Nose and Throat Hospital of Fudan University, Shanghai 200031, China; Department of Ophthalmology, Casey Eye Institute, Oregon Health & Science University, Oregon, USA

**Keywords:** gap junction, connexin43, TGF beta1, mechanical stretch, actin

## Abstract

Many physiological and pathophysiological processes in cells or tissues are involved in mechanical stretch, which induces the gap junction gene expression and cytokine TGF beta changes. However, the underlying mechanisms of the gap junction gene expression remain unknown. Here, we showed that the mRNA and protein levels of Cx43 in human umbilical vein endothelial cells (HUVECs) were significantly increased after 24 h stretch stimulation, and TGF beta1 (not TGF beta2) expression was also upregulated. Administration of TGF beta1 into HUVECs without stretch also induced upregulation of Cx43 expression. However, SB431542, a specific inhibitor of the TGF beta1 receptor, blocked the Cx43 protein upregulation caused by TGF beta1. Further, the increase of Cx43 protein expression under the stretch condition was partially blocked by SB431542; it was also partially blocked by simultaneous administration of anti-TGF beta1 monoclonal neutralization antibody. Importantly, the upregulation of Cx43 induced by stretch was blocked by the administration of actin and microtubule inhibitors, while NEDD4, a key element in mediating Cx43 protein ubiquitination and degradation, was not changed under the stretch condition. In conclusion, upregulation of Cx43 expression under the 24 h stretch condition is mediated via TGF beta1 receptor signaling pathway, and it also involves the actin and microtubule cytoskeletal network.

## 1 Introduction

Cells or tissues in normal pathophysiological conditions undergo stretch to maintain homeostasis. In blood vessel smooth muscle cells, mechanical stretch is a physiological feature that occurs by regulating blood pressure and blood flow. After a meal, the stomach is expanded and undergoes mechanical stretch for fulfilling its normal digestive function [1].

Gap junctions are intercellular channels that allow the direct cell to cell exchange of ions and small molecules with molecular weight less than 1,000 Da such as water, ATP, calcium, CAMP, CGMP, IP3, etc. Gap junctional intercellular communications play pivotal roles in maintaining the physiological function of cells and tissues, which include cell differentiation, proliferation, survival, apoptosis, and tissue homeostasis [2]. There are 21 connexin genes in the human genome and, among these connexins, connexin43 (Cx43) is the most widely distributed gap junction protein in different cells [3].

Transforming growth factor beta1 (TGF beta1) is a secreted polypeptide cytokine and it belongs to the transforming growth factor superfamily [4]. TGF beta1 signaling pathway is involved in many important cellular processes such as cell development, proliferation, and differentiation in a variety of cell types [5], and is the same as Cx43.

Relevant literature studies indicate that either TGF beta1 or Cx43 is increased in different cell types under different stretch conditions. For example, cyclic stretching showed selectively upregulation of TGF beta1 but not TGF beta2 in cultured rat mesangial cells [6]. Mechanical stretch increased the TGF beta1 expression in vascular smooth muscle cells, fetal human intestinal smooth muscle cells, cardiomyocytes, and hepatic stellate cells [7,8,9,10]. In cultured neonatal rat cardiomyocytes, cyclic mechanical stretch showed a significant upregulation of Cx43 expression under 4–16 h stretch [11]. In human trabecular meshwork cells, it has been reported that Cx43 protein expression was increased under the mechanical stretch condition [12]. Recently, some studies showed that the administration of exogenous TGF beta1 into cultured cells can modify Cx43 expression [13,14,15,16,17,18].

Taking into consideration the similar functions of Cx43 and TGF beta1, it seems possible that TGF beta1 and Cx43 have a functional correlation in some cell types. To date, the effect of TGF beta1 on Cx43 expression under the stretch condition has not yet been investigated. Therefore, in the current study, we try to evaluate Cx43 and TGF beta1 expression under the stretch condition, and the effect of the TGF beta1 signaling pathway on Cx43 expression in cultured human umbilical-vein endothelial cells (HUVECs). In addition, Cx43 has been demonstrated to be associated with the actin and microtubule cytoskeleton network [19,20,21]. Cx43 has also been shown to be heavily regulated by an E3 ubiquitination enzyme, namely NEDD4 [22]. Therefore, we also investigated the effect of the actin and microtubule cytoskeletal network, and NEDD4 on mechanical stretch-induced Cx43 expression changes.

## 2 Materials and methods

### 2.1 Materials and reagents

Rabbit polyclonal anti-Cx43 antibody (Cat. No 71-0700) was purchased from Invitrogen (CA, USA). N-terminal monoclonal anti-Cx43 antibody and monoclonal anti-NEDD4 antibody were obtained from BD Bioscience (NJ, USA). Polyclonal anti-GAPDH and anti-beta tubulin were acquired from Sigma Aldrich (MO, USA). Anti-Cx37 antibody, SB431542, monoclonal antibodies for TGF beta1 and TGF beta2, latrunculin A, and nocodazole were obtained from Invitrogen, Tocris (UK), BD Bioscience, and Sigma-Aldrich, respectively.

### 2.2 Cell culture and treatment

Human umbilical endothelial cells (HUVECs) were purchased from the American Tissue and Cell Culture (ATCC, MD, USA) and cultured in an F-12K cell culture medium containing 10% fetal bovine serum (FBS), 1% penicillin, and 0.03 mg/mL endothelial cell growth supplement. After 5 h stabilization, cells underwent 12 or 24 h mechanical stretch, and then they were collected for subsequent experiments. Control cells were cultured under the same condition in collagen I-coated Bio flex 6-well plates without mechanical stretch.

For a mechanical stretch, HUVECs were switched to serum-free DMEM for 5 h, followed by a cyclic mechanical stretch for 24 h with the following parameters: 10% stretching, 1 cycle/s using the FX-5000 Tension System (Flexcell, NC, USA). In addition, cells were incubated with 50 µM latrunculin A (actin cytoskeleton inhibitor) or/and nocodazole (microtubule inhibitor) that were dissolved in dimethyl sulfoxide (DMSO). DMSO administration was used as the control and then subjected to mechanical stretch treatment.

### 2.3 Enzyme-linked immunosorbent assay (ELISA)

The levels of TGF beta1 and TGF beta2 in HUVECs were measured as previously described [23]. Briefly, after a 24 h mechanical stretch, TGF beta1 and TGF beta2 concentrations in HUVECs were measured using ELISA kits with standard protocols provided by the manufacturer (R&D Systems, MN, USA).

### 2.4 Total RNA extraction, RT-PCR, and real-time PCR

RNA extraction, RT-PCR, and real-time PCR were performed according to the previous description and the manufacturer’s protocol [20]. Briefly, cells were collected after 12 or 24 h mechanical stretch. Total RNA of the cells was extracted using TRIzol reagents (Invitrogen, CA, USA) and determined using a NanoDrop 3300 Fluorospectrometer (Thermo Fisher Scientific, MA, USA). Reverse transcription was conducted using SuperScript® III reverse transcriptase and oligo(dT)20 primer (Invitrogen, CA, USA). The PCR conditions for annealing were dependent on the primers. The single-band PCR product was verified by electrophoresis in a 2% agarose gel stained with ethidium bromide. Real-time PCR was performed using RT² SYBR Green Fluor qPCR Mastermix (QIAGEN, Germany) according to the standard protocol.

The following primers were used in this study.

GAPDH sense: 5′-AATCCCATCACCATCTTCCAGGAG-3′

GAPDH antisense: 5′-CACCCTGTTGCTGTAGCCAAATTC-3′

Cx37 sense: 5′-TCAGCACACCCACCCTGGTCT-3′

Cx37 antisense: 5′-GGATGCGCAGGCGACCATCTT-3′

Cx43 sense: 5′-GGTCTGAGTGCCTGAACTTGCCT-3′

Cx43 antisense: 5′-AGCCACACCTTCCCTCCAGCA-3′

TGF-beta1 sense: 5′-CCCAGCATCTGCAAAGCTC-3′

TGF-beta1 antisense: 5′-GTCAATGTACAGCTGCCGCA-3′

TGF-beta2 sense: 5′- GAAGACCCCACATCTCCTGCTA-3′

TGF-beta2 antisense: 5′-AGCAATAGGCCGCATCCAA-3′.

### 2.5 Human Cx43 expression vector construction

The procedure for constructing a human Cx43 expression vector was conducted as previously described [24]. Oligonucleotide primers were obtained from Integrated DNA Technologies (IDT) Inc. (Coralville, IA, USA), and transfection reagents were purchased from Life Technologies Inc. (MA, USA). The primers chosen for RT-PCR were based on the human Cx43 sequence (NCBI Reference Sequence: NM_000165.5). These were full-length Cx43 sense primer 5′-ATGGGTGACTGGAGCGCCTTAG-3′ and full-length Cx43 antisense primer 5′-CTAGATCTCCAGGTCATCAGG-3′.

For amplification of human Cx43 coding cDNA, the PCR was conducted in 20 μL of a solution containing 2 μL of 10× PCR buffer, 0.8 μL of 50 mM MgCl_2_, 200 μM dNTP, 100 ng of sense and antisense primers,1 unit of Taq DNA polymerase, and 1 μL of template cDNA. For the PCR, DNA was denatured at 95°C for 3 min, and then subjected to 30 cycles at 95°C for 60 s, 55°C for 60 s, and 72°C for 90 s, followed by a final extension at 72°C for 10 min for T-A cloning. PCR products were separated by electrophoresis in 1% agarose gel, stained with ethidium bromide, and purified using a gel purification kit (QIAGEN, Germany). PCR products were subcloned into pCR 2.1 vector, digested with *Bam*HI and *Apa*I, and ligated into pcDNA3.1 expression vector using T4 DNA ligase according to the manufacturer’s instructions (Invitrogen, CA, USA). Recombinant plasmids were extracted and verified with *Kpn*I and *Bst*X1 digestion. At least two recombinant plasmids were sequenced using the T7 universal primer and specific Cx43 primer for confirmation.

HeLa cells (ATCC, MD, USA) were grown in Dulbecco’s modified Eagle’s medium with low glucose content and supplemented with 10% FBS and 1% penicillin/streptomycin. For transient transfection, HeLa cells with 80% confluence were transfected for 48 h with pcDNA3.1 vector, full-length Cx43-pcDNA3, or truncated Cx43-pcDNA3.1 plasmids using Lipofectamine 3000 reagent (Thermo Fisher Scientific, MA, USA) as described previously [24,25]. Cells were harvested followed by a western blotting assay for validating anti-Cx43 antibody specificity.

### 2.6 Immunofluorescence labeling

The procedure for immunofluorescence labeling was used as previously described [24,26]. Briefly, mechanical stretched and nonstretched HUVECs grown in 6-well plates were fixed with 2% cold paraformaldehyde for 10 min, and then were washed with PBS. For immunolabeling, slides were incubated in 50 mM tris-HCl, pH 7.4, containing 1.5% sodium chloride (TBS), 0.3% Triton X-100 (TBSTr), and 5% NGS for 24 h at 4℃ with Cx43 polyclonal antibody at 1:1,000, which were then washed for 1 h in TBSTr and incubated for 1.5 h at room temperature simultaneously with Alexa Fluor 488-conjugated goat anti-rabbit IgG at 1:1,500 dilution (Molecular Probes, OR, USA). Following incubation with secondary antibodies, slides were sequentially washed with TBSTr for 20 min and with 50 mM tris-HCl buffer, pH 7.4, for 30 min. Confocal immunofluorescence images were gathered using a confocal microscope using the Fluoview program (Olympus, Japan).

### 2.7 Western blotting

The procedure for western blotting was as described previously [24,27]. Briefly, cells under mechanical stretch and nonstretch conditions were harvested using an IP buffer (20 mM tris-HCl, pH 8.0, 140 mM NaCl, 1% Triton X-100, 10% glycerol, 1 mM EGTA, 1.5 mM MgCl_2_, 1 mM dithiothreitol, 1 mM phenylmethylsulfonyl fluoride, and 5 µg/mL each of leupeptin, pep-statin A, and aprotinin) and sonicated. Homogenates were centrifuged at 20,000×*g* for 20 min at 4°C and were determined using Bradford reagent (Bio-Rad, CA, USA). Proteins containing 5% β-mercaptoethanol were boiled for 5 min and were separated by 10% SDS-PAGE (5 µg of protein per lane), followed by transblotting to polyvinylidene difluoride membranes (Bio-Rad, CA, USA) in standard tris-glycine transfer buffer, pH 8.3, containing 0.5% SDS. Membranes were blocked for 2 h at room temperature in TBSTw (10 mM tris-HCl, pH 8.0, 150 mM NaCl, and 0.2% Tween 20) containing 5% nonfat milk powder, rinsed briefly in TBSTw, and incubated overnight at 4°C with polyclonal anti-Cx43 primary antibody diluted at 1:1,000 in PBS containing 1% nonfat milk or polyclonal anti-alpha-GAPDH antibody diluted at 1:1,000 (Novus Biologicals, CO, USA) in TBSTw containing 1% nonfat milk powder. Membranes were washed four times in TBSTw for 40 min, incubated with horseradish peroxidase-conjugated donkey anti-rabbit IgG or anti-mouse IgG diluted 1:5,000 (Sigma-Aldrich, MO, USA) in TBSTw containing 1% nonfat milk powder, washed four times in TBSTw for 40 min, and resolved using an Odyssey® CLx Infrared Imaging System (LI-COR, NE, USA). The optical density (OD) of the specific band was also acquired.

### 2.8 Statistical analysis

Each experiment was performed in triplicate. All data were expressed as mean ± standard error of the mean (SEM). The one-way ANOVA test was employed to examine the statistically significant differences between groups. Significant differences were set at *P < 0.05, **P < 0.01, and ***P < 0.001.


**Ethical approval:** The conducted research is not related to either human or animal use.

## 3 Results

### 3.1 Upregulation of Cx43 mRNA and protein under the stretch condition

**Figure 1:**
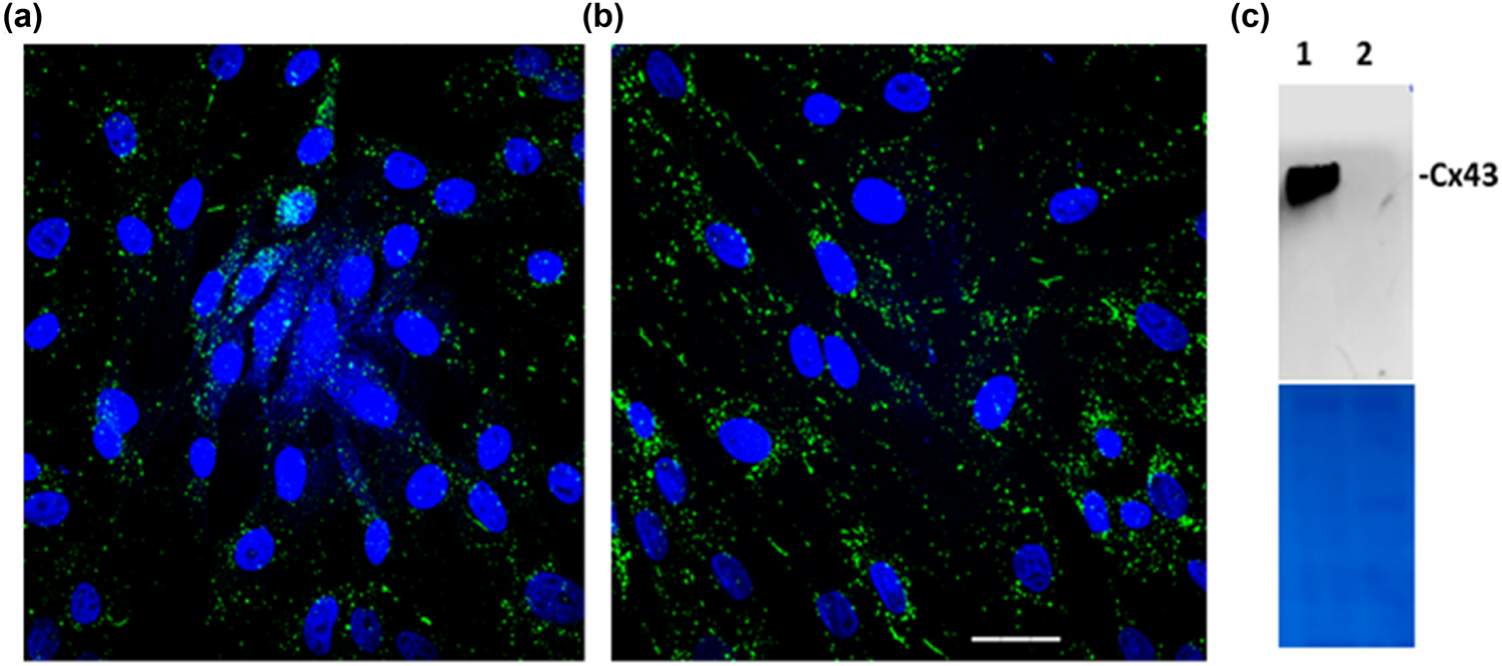
Upregulation of Cx43 expression in HUVECs under stretch. (a) Cx43 expression (green) in HUVECs with nonstretch. (b) Cx43 expression in HUVECs with 24 h stretch. Scale bar = 20 µm. (c) Validation of anti-Cx43 specificity in HeLa cells by western blotting. (1) HeLa cells were transfected with a Cx43 expression vector (Cx43-pcDNA3.1). (2) HeLa cells were transfected with an empty vector (pcDNA3.1). Coomassie blue staining was used as a protein loading control.

First, we identified the specificity of the anti-Cx43 antibody in HeLa cells. As shown in Figure 1c, Cx43 protein was expressed in HeLa cells transfected with Cx43-pcDNA3.1 vector and not cells transfected with empty vector. Further, the anti-Cx43 antibody was used to validate the Cx43 expression in HUVECs. As shown in Figure 1a and b, Cx43 protein showed higher expression in HUVECs under the 24 h stretch condition than that with the nonstretch condition.

**Figure 2:**
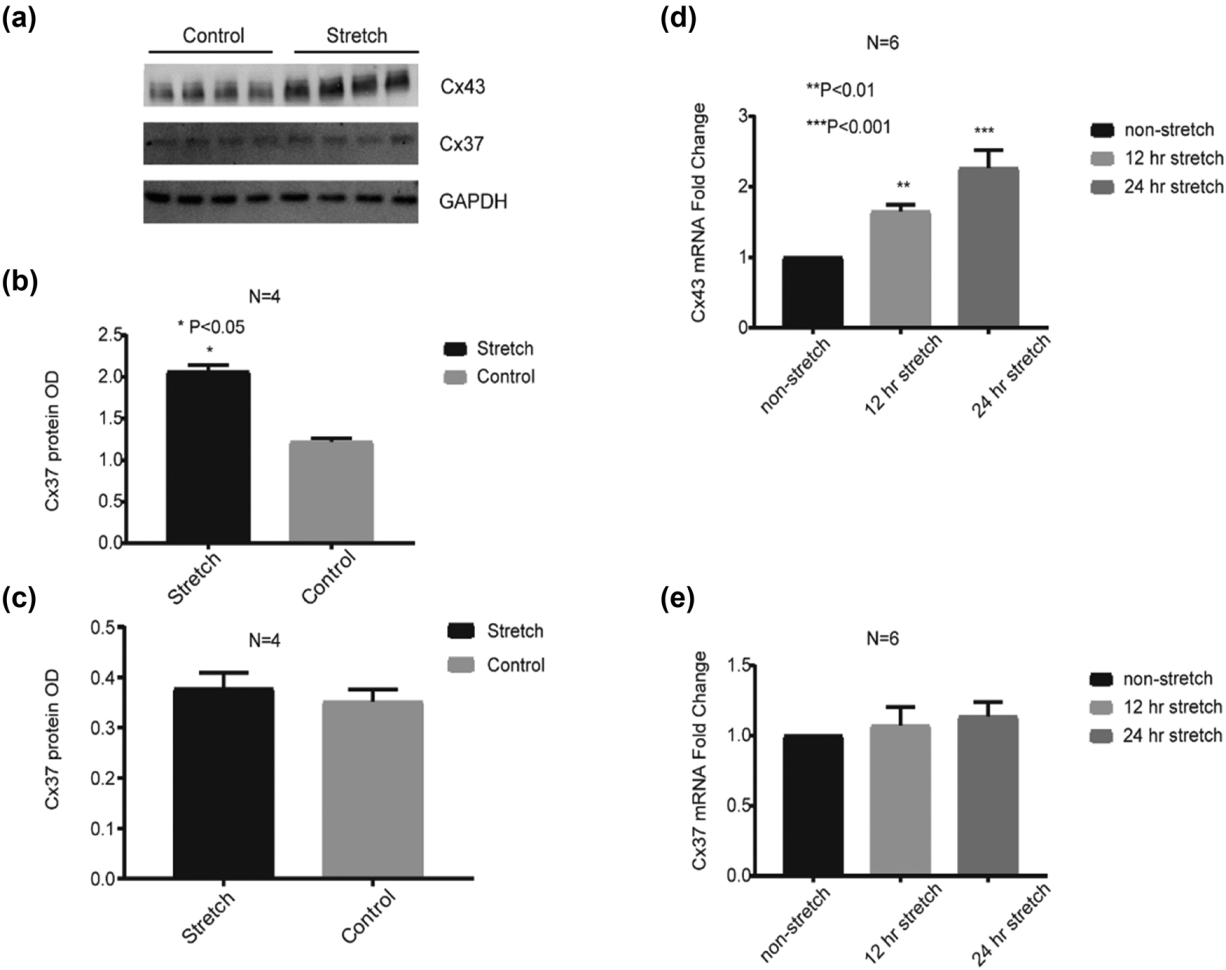
Increased Cx43 protein and mRNA expression in HUVECs after stretch. (a–c) The relative protein levels of Cx43 and Cx37 in HUVECs under 24 h stretch. (d and e) The relative mRNA expression of Cx43 and Cx37 in HUVECs under 12 and 24 h stretch. *P < 0.05, **P < 0.01, and ***P < 0.001 vs the control or the nonstretch.

The immunofluorescence labeling of Cx43 showed typical Cx43 cellular localization, but the more accurate quantification of Cx43 at mRNA and protein levels needs real-time qPCR and western blotting techniques. Subsequently, we investigated Cx43 and Cx37 expression in mRNA and protein levels under the 12 or 24 h stretch condition. As shown in Figure 2a–c, western blot results showed that Cx43 protein was significantly increased after 24 h stretch (*P* < 0.05), while there was no change in the Cx37 protein level in the stretch and nonstretch conditions. Real-time qPCR was used to determine Cx43 and Cx37 mRNA expression under the stretch condition. As shown in Figure 2d and e, Cx43 mRNA was significantly increased after 12 or 24 h stretch (*P* < 0.01); however, Cx37 mRNA expression did not show statistically significant changes under stretch and nonstretch conditions. It appears that stretch can increase Cx43 mRNA and protein levels, but no changes in Cx37 mRNA and protein levels.

### 3.2 Increased TGFbeta1 mRNA and protein levels after 24 h stretch

**Figure 3:**
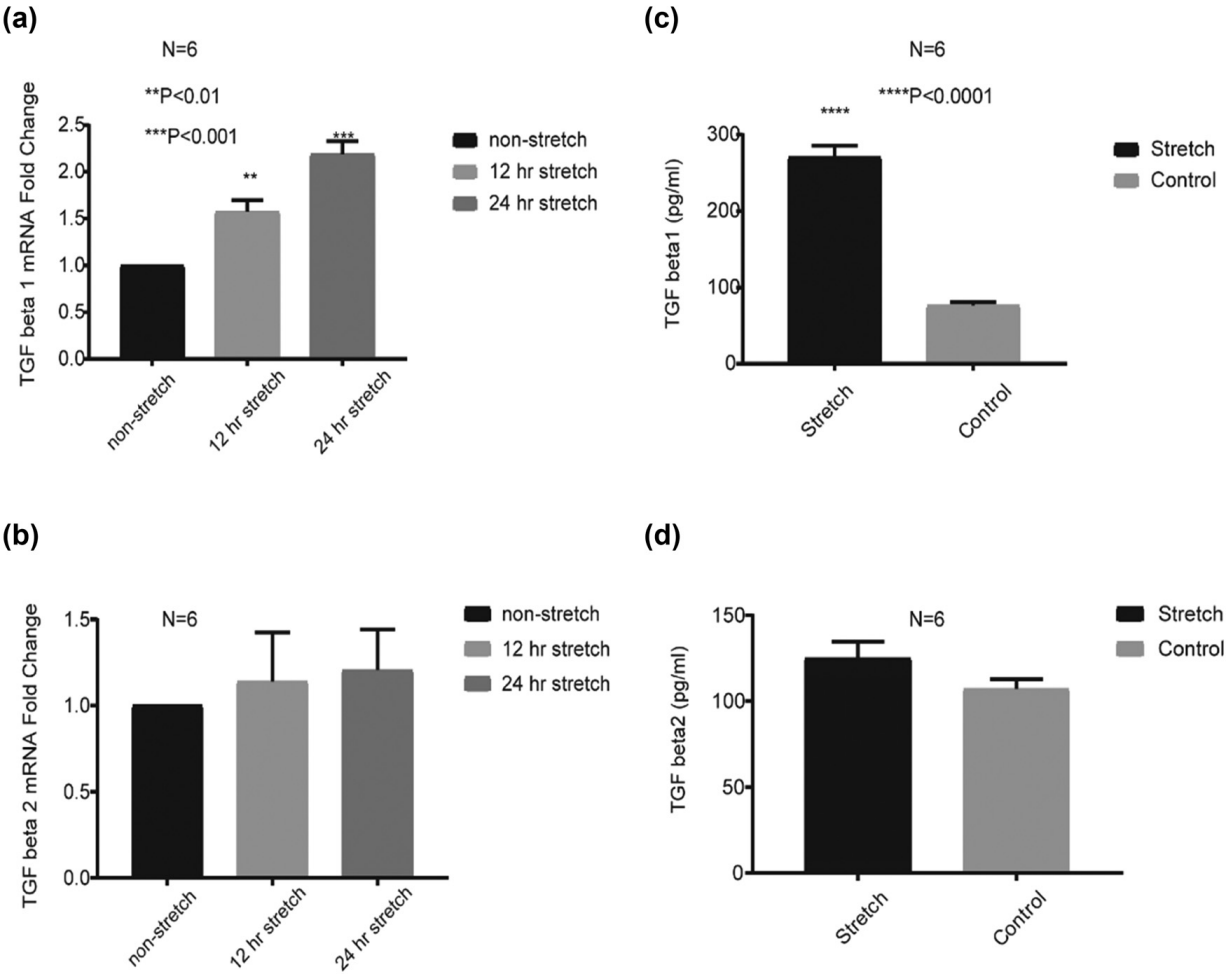
The increased TGF beta1 expression in HUVECs after stretch. (a and b) The relative mRNA expression of TGF beta1 and TGF beta2 in HUVECs with 12 and 24 h stretch. (c and d) The levels of TGF beta1 and TGF beta2 in HUVECs under 24 h stretch measured by enzyme-linked immunosorbent assay. **P < 0.01, ***P < 0.001, and ****P < 0.0001 vs the nonstretch or the control.

In the next step, we carried out a detailed analysis of TGFbeta1 and TGFbeta2 mRNA levels under 12 and 24 h stretch conditions using real-time qPCR and ELISA techniques. As shown in Figure 3a and b, the TGFbeta1 mRNA level was increased with statistical significance under the 12 or 24 h stretch condition (*P* < 0.01); however, there was no change of the TGF beta2 mRNA expression level under the same condition. From this analysis, it is clearly evident that TGF beta1 was increased under the stretch condition. In addition, the TGF beta1 protein concentration in the cell culture medium was significantly higher compared with the cell culture medium under the no stretch condition (*P* < 0.0001, Figure 3c). However, for TGF beta2 concentration, there was no statistical difference between stretch and no stretch conditions (Figure 3d).

### 3.3 Effects of TGF beta1 and its receptor inhibitor on Cx43 expression

**Figure 4:**
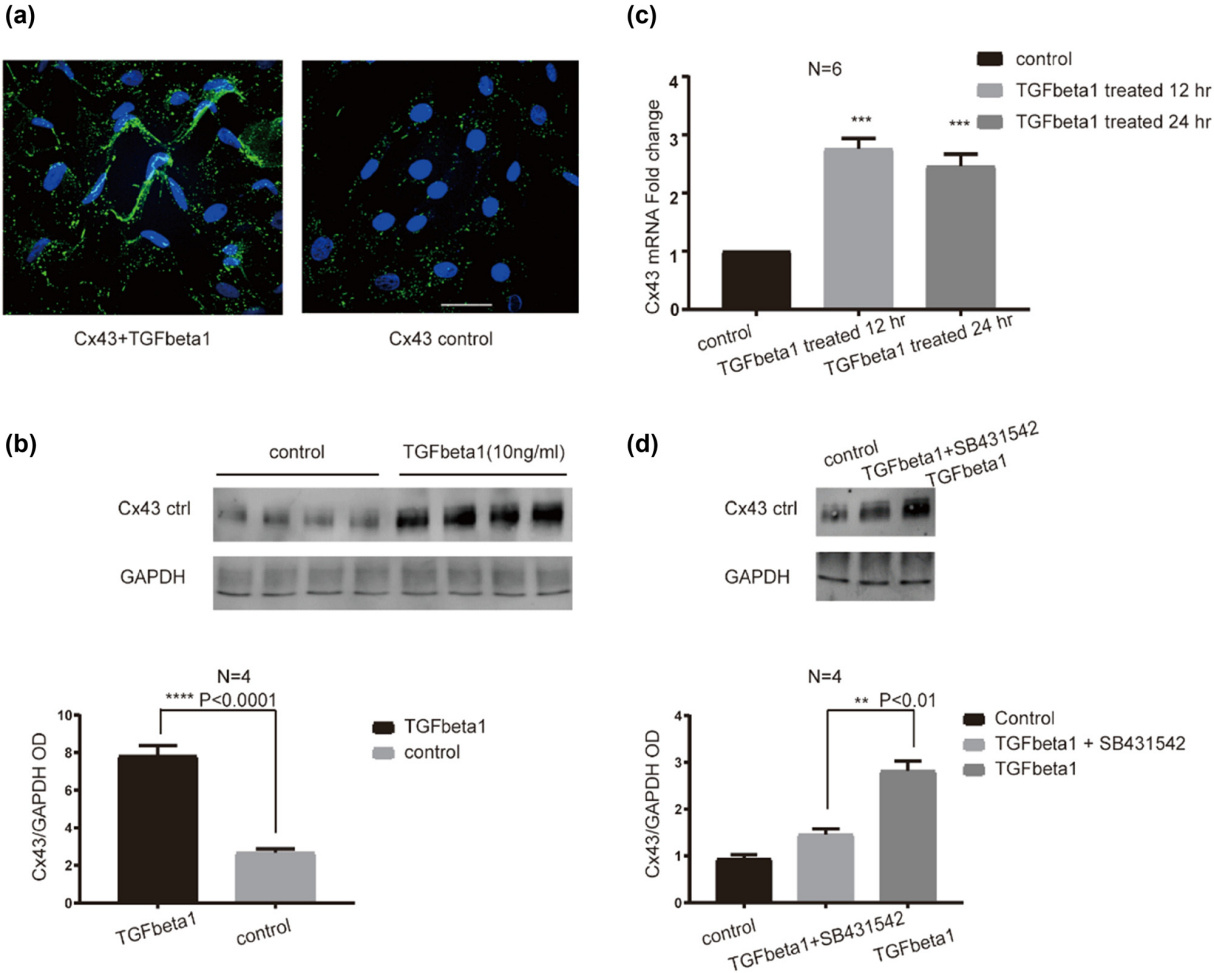
TGF beta1 administration increased the Cx43 expression in HUVECs. (a) The expression of Cx43 (green) in HUVECs after TGF beta1 treatment by immunofluorescence staining. Scale bar = 20 µm. (b) The relative protein level of Cx43 in HUVECs after TGF beta1 treatment by western blotting. (c) The relative mRNA expression of Cx43 in HUVECs after TGF beta1 treatment for 12 and 24 h by real-time RT-PCR. ***P < 0.001 and ****P < 0.0001 vs the control. (d) The relative protein expression of Cx43 in HUVECs after TGF beta1 or/and SB431542 (a TGF beta1 receptor inhibitor) treatment. **P < 0.01 between the TGF beta1 and the TGF beta1 + SB431542.

It has been reported that TGF beta1 expression can regulate Cx43 expression in diverse cells. Therefore, we investigated whether TGF beta1 can increase or decrease Cx43 expression in HUVECs. As shown in Figure 4a, administration of TGF beta1 (10 ng/mL) into HUVECs for 24 h increased Cx43 labeling. Western blot results showed strong Cx43 detection after TGF beta1 treatment compared with control cells (*P* < 0.0001, Figure 4b). Real-time qRT-PCR indicated that TGF beta1 significantly upregulated the Cx43 mRNA expression (*P* < 0.001, Figure 4c). Furthermore, we used a specific TGF beta1 receptor inhibitor (SB431542) to check whether it can block the upregulation of Cx43 expression induced by TGF beta1. As shown in Figure 4d, Cx43 protein expression was significantly inhibited after simultaneously treating cells with TGF beta1 (10 ng/mL) and SB431542 (5 µM) (*P* < 0.01). In summary, these data demonstrated that TGF beta1 can increase Cx43 mRNA and protein expression in HUVECs, and it appears that the effect of TGF beta1 on Cx43 expression is via TGF beta1 receptor.

### 3.4 Upregulation of Cx43 under the stretch condition can be partially blocked by SB431542, an inhibitor of TGF beta1 receptor

**Figure 5:**
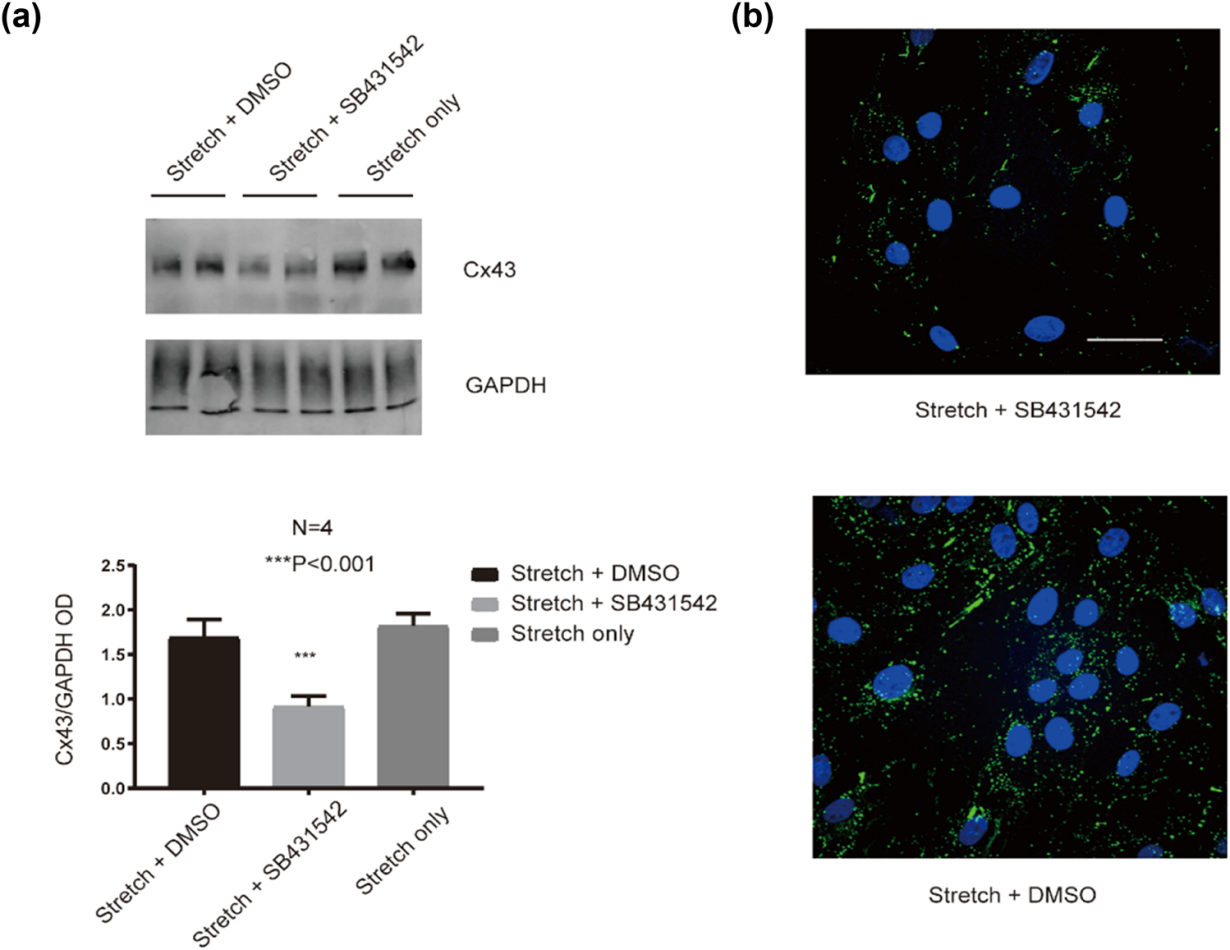
SB431542 blocks the upregulation of Cx43 in HUVECs under the stretch condition. (a) The relative protein level of Cx43 in HUVECs with stretch or/and SB431542 treatment. ***P < 0.001 vs the stretch + DMSO. (b) Cx43 expression in HUVECs with stretch or/and SB431542 treatment by immunofluorescence staining. Scale bar = 20 µm.

In order to test whether SB431542 can regulate Cx43 expression in HUVECs under the stretch condition, HUVECs transfected with Cx43 were treated with SB431542 (5 µM) under the 24 h stretch condition. As shown in Figure 5a, the results showed that the upregulation of the Cx43 protein level was significantly reduced in HUVECs co-cultured with SB431542 (5 µM) under the 24 h stretch condition compared with HUVECs co-cultured with DMSO, a reagent used for dissolving SC431542 under 24 h stretch only (*P* < 0.001). Similarly, the immunofluorescence labeling of Cx43 was also reduced after administration of SB431542 (5 µM) under the stretch condition (Figure 5b). These results clearly indicated that administration of TGF beta1 receptor inhibitor, SB431542, to HUVECs under the stretch condition can partially block the effect of stretch-induced Cx43 protein expression.

### 3.5 Upregulation of Cx43 under the stretch condition can be partially blocked by TGF beta1 monoclonal neutralization antibody

**Figure 6:**
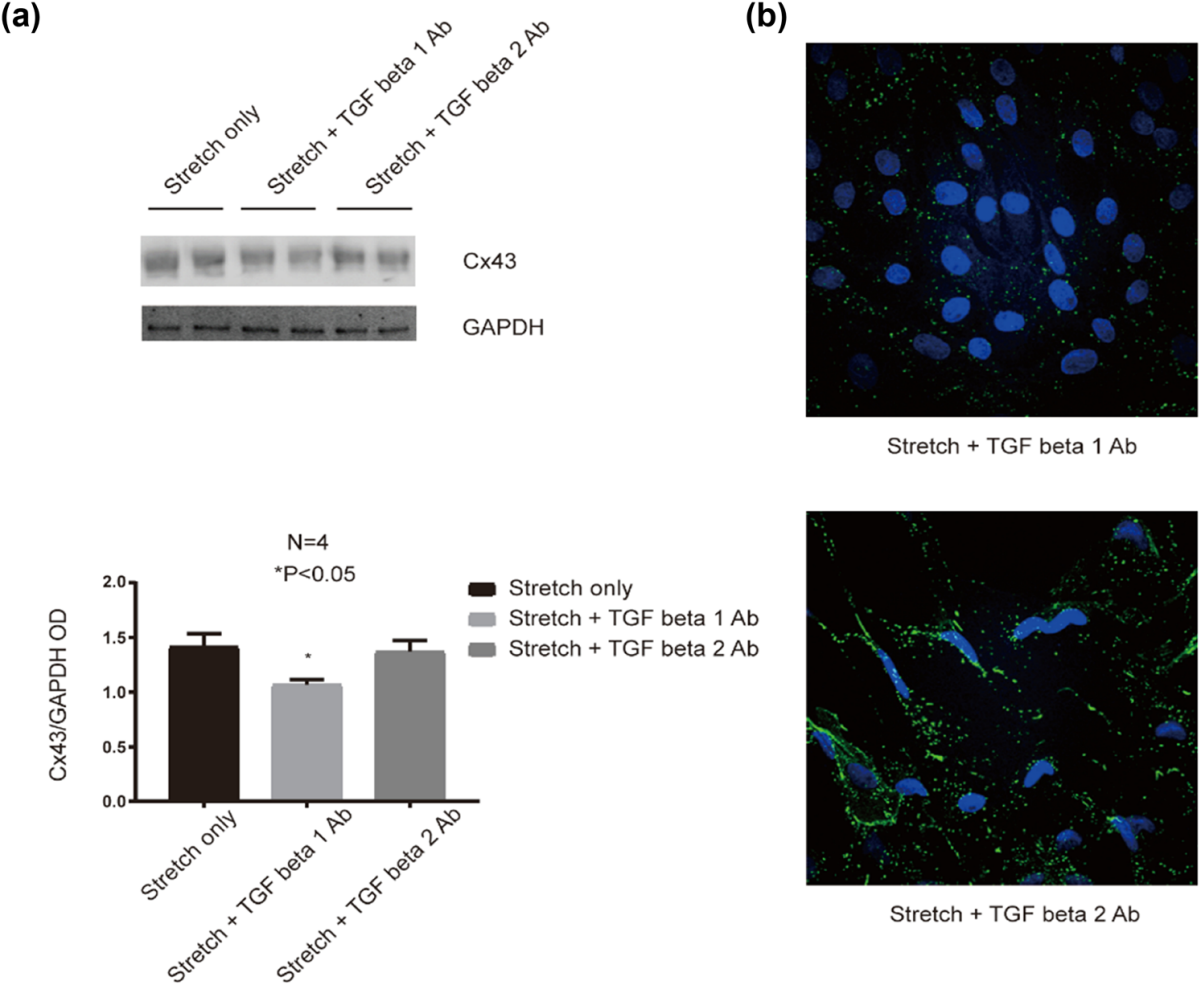
Administration of TGF beta1 neutralizing antibody (Ab) blocks the increment of Cx43 expression in HUVECs with stretch. (a) The relative protein level of Cx43 in HUVECs with stretch or/and TGF beta1 Ab/TGF beta2 Ab by western blotting. *P < 0.05 vs the stretch only. (b) Cx43 expression in HUVECs with stretch or/and TGF beta1 Ab/TGF beta2 Ab by immunofluorescence staining. Scale bar = 20 µm.

It is well known that administration of monoclonal anti-TGF beta1 antibody can block the TGF beta1 biological activity; therefore, we checked the Cx43 expression after administration of TGF beta1 monoclonal neutralization antibody into HUVECs under the 24 h stretch condition. As shown in Figure 6a and b, the Cx43 protein level was significantly reduced in HUVECs co-cultured with anti-TGF beta1 monoclonal neutralization antibody (5 µg/mL) under the stretch condition compared with HUVECs under the stretch condition only (*P* < 0.05). Both the western blot and immunofluorescence data indicated that administration of TGF beta1 monoclonal neutralization antibody to HUVECs under the stretch condition can partially block the upregulation of Cx43 expression under the 24 h stretch condition.

### 3.6 Upregulation of Cx43 under the stretch condition can be partially blocked by the administration of actin and microtubule cytoskeleton inhibitors

**Figure 7:**
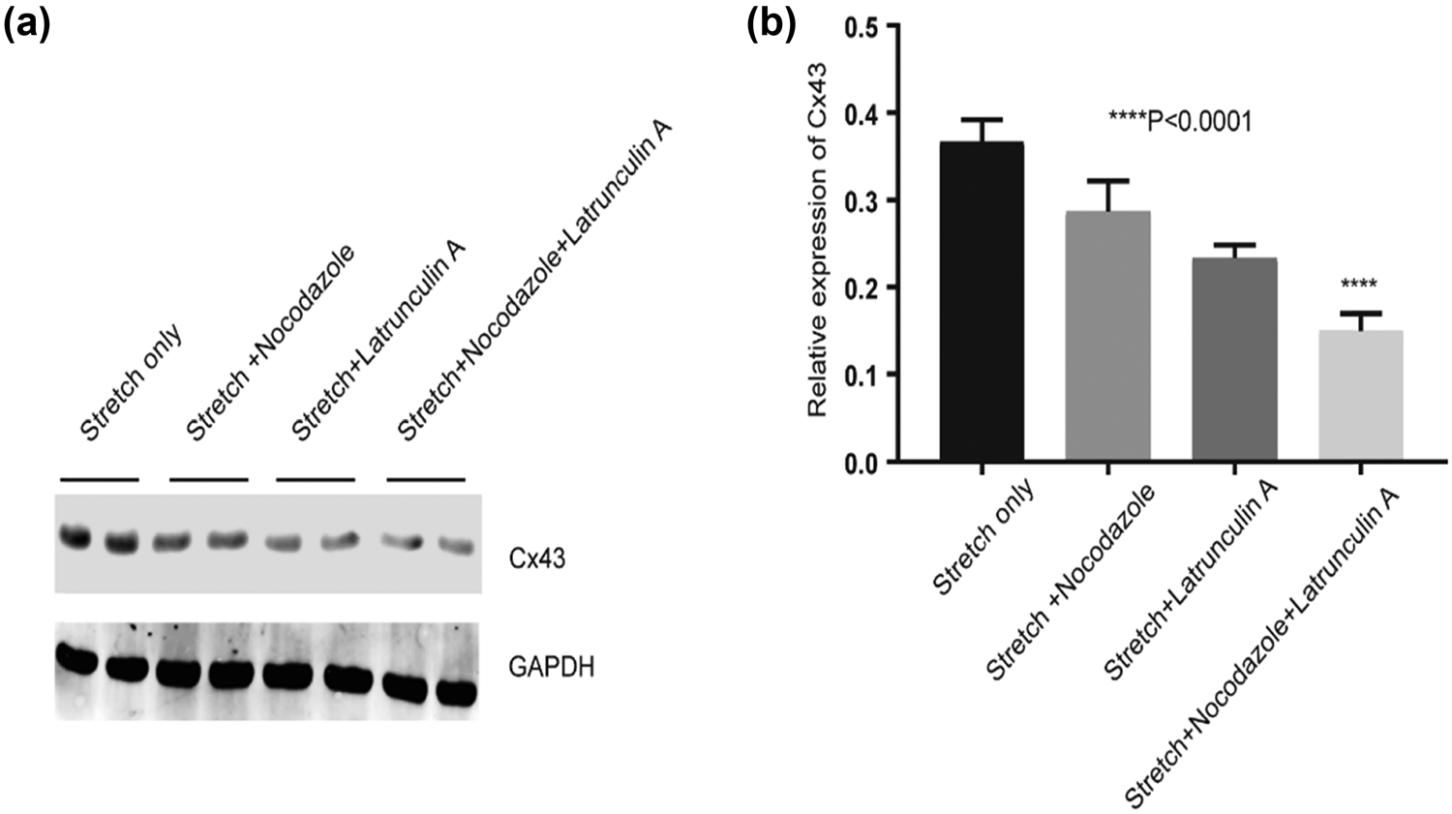
Administration of actin and microtubule cytoskeleton inhibitor blocks the increment of Cx43 expression in HUVECs with stretch. (a and b) The relative protein level of Cx43 in HUVECs with stretch, nocodazole, or/and latrunculin A by western blotting. ****P < 0.0001 vs the stretch only.

We further investigated whether the upregulation of Cx43 under stretch is involved in the actin and microtubule tubulin cytoskeleton. Immunoblot results showed that the increment of Cx43 protein expression under mechanical stretch was partially inhibited in stretched HUVECs after administering actin cytoskeleton inhibitor latrunculin A (1 µM) or microtubule inhibitor nocodazole (1 µM), and after administering latrunculin A (1 µM) and nocodazole (1 µM) simultaneously; it appears that stretch-induced upregulation of Cx43 was further dramatically inhibited (*P* < 0.0001, Figure 7a and b). These data indicated that both actin and microtubule cytoskeleton were involved in the upregulation of Cx43 expression under the mechanical stretch condition.

### 3.7 No change in NEDD4 protein expression under the stretch condition

**Figure 8:**
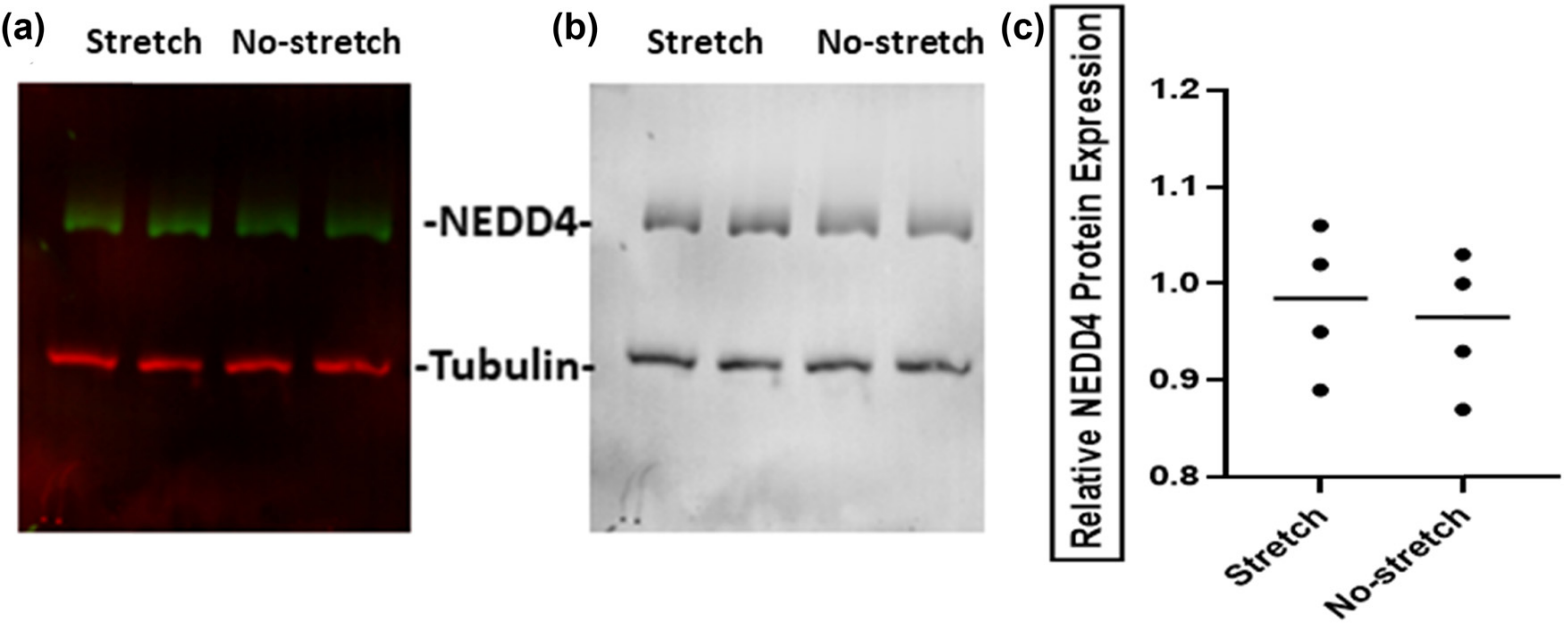
NEDD4 expression in HUVECs with stretch. (a–c) The relative protein level of NEDD4 in HUVECs with stretch by western blotting.

To date, NEDD4 (neuronal precursor cell-expressed developmentally downregulated 4), an E3 ubiquitin ligase, has been shown to interact with Cx43 and promote Cx43 protein degradation via endocytosis and lysosomal sorting. The previous work has shown that there is endogenous NEDD4 expression in HUVECs, so we investigated the NEDD4 protein level in HUVECs under the stretch condition. Western blotting showed that NEDD4 protein bands were observed in HUVECs both under stretch and no-stretch conditions (Figure 8a and b). The statistical results showed that there was no significant difference in NEDD4 protein expression in HUVECs under stretch and no-stretch conditions (Figure 8c), indicating NEDD4 may not play an important role in the upregulation of Cx43 expression under the mechanical stretch condition.

## 4 Discussion

Mechanical stretch plays an important role in sustaining myofibroblast phenotype and function in microtissues, and homeostasis [28]. Cx43, a gap junction protein, is involved in the regulation of homeostasis under mechanical stretch [29]. In this study, we found that stretch stimulation promoted the Cx43 expression in HUVECs. The expression of TGF beta1 was also upregulated in HUVECs after stretch stimulation. Moreover, administration of TGF beta l enhanced the Cx43 expression in HUVECs, whereas SB431542 (a TGF beta1 receptor inhibitor) or anti-TGF beta1 Ab addition reversed this result. SB431542 and anti-TGF beta1 Ab can also block the increased Cx43 expression in HUVECs after stretch. Furthermore, the administration of actin and microtubule cytoskeleton inhibitors downregulated the increment of Cx43 expression in HUVECs after stretch. Therefore, we conclude that the effect of stretch upregulating the Cx43 expression is mediated by the TGF beta1 receptor signaling pathway, as well as actin and microtubule cytoskeletal networks.

Cx43 is one of the most ubiquitously distributed gap junction proteins, which plays a pivotal role in the regulation of human homeostasis, including cell differentiation, growth, and apoptosis. Mechanical stretch is capable of regulating Cx43 expression at both mRNA and protein levels. Previous literature studies showed that stretch and shear stress can increase Cx43 expression at different cell types and different stretch systems [29]. Previous works also showed that mechanical stretch can change the TGF beta level [6,7,8,9,10]. Our results showed that stretch upregulated the expression of Cx43 and TGF beta1 in HUVECs, which is in line with previous works [6,7,8,9,10]. However, previous works did not investigate the mechanism of stretch on both Cx43 and TGF beta expression. The TGF beta signaling pathway includes a canonical pathway via Smad2/3 signaling and a noncanonical signaling pathway via P38 signaling [5]. Previous works showed that TGF beta1 is an important regulator for Cx43 expression in different cell lines or tissues [13,14,30,31]. They also showed that the TGF beta1 may regulate Cx43 expression in a broad manner with a diversity of cells. To the best of our knowledge, our work demonstrates the possible mechanism of stretch-induced upregulation of Cx43 expression, which is mediated by TGF beta1.

The cellular cytoskeleton plays an important role in maintaining the homeostasis of cells and tissues, and many cellular processes and mechanical stretch can affect the actin cytoskeleton [32–34]. Cx43 has been reported to be associated with both actin and microtubule cytoskeleton [35,36]. Blocking actin cytoskeleton and microtubule network by latrunculin A can retard gap-junctional intercellular communication (GJIC) in cultured astrocytes [37] and stop trafficking Cx43 to the plasma membrane [5,38]. Our work showed that the upregulation of Cx43 in HUVECs induced by mechanical stretch is blocked by actin and microtubule cytoskeleton inhibitors, in particular, by combined treatment. These data indicated that the actin cytoskeleton and microtubule cytoskeleton were involved in the regulation of Cx43 expression in HUVECs under the mechanical stretch condition.

The removal and disposal of Cx43 are mediated via ubiquitination-dependent and ubiquitination-independent pathways [22], which involved a key E3 ubiquitin ligase, NEDD4 [39]. NEDD4 interacts with Cx43 through the WW domain of NEDD4 and a PY consensus motif at the C-terminal Cx43; hence, there were no changes in NEDD4 expression in HUVECs under the stretch condition. Therefore, we conclude that NEDD4 is not involved in the Cx43 expression changes under the stretch condition. However, a recent report demonstrated that the NEDD4 protein level was significantly increased in breast cancer samples with the downregulated Cx43 expression, indicating that the reduced Cx43 expression may be related to increased NEDD4 expression [40,41].

It is noteworthy to mention that Cx43 can regulate the TGF beta signaling pathway via its competition with SMAD2 for binding to microtubules [31,42]. In our cell culture system, whether upregulated Cx43 can play a role in modulating TGF beta1 upregulation remains unknown. Further, there are additional growth factors other than TGF beta1 that can regulate Cx43 expression, such as epidermal growth factor (EGF) and vascular endothelial growth factor (VEGF)[43,44]. In addition, Cx43 expression can be regulated at the transcriptional level or posttranslational level such as changing degradation. Our results demonstrated that stretch can upregulate Cx43 mRNA in HUVECs; however, whether changes in Cx43 degradation under the stretch condition is also an important contributor to Cx43 protein upregulation need further investigation.

In summary, our current study demonstrates that mechanical stretch upregulates both Cx43 and TGF beta1 expression in HUVECs, and the upregulation of Cx43 expression under the stretch condition can be partially blocked by the TGF beta1 receptor inhibitor SB431542 or by specific anti-TGF beta1 Ab, indicating the involvement of TGF beta1 signaling pathway in the regulation of Cx43 expression in HUVECs under the stretch condition. More importantly, we showed that the upregulation of Cx43 induced by mechanical stretch can be blocked by the administration of actin and microtubule cytoskeleton inhibitors, indicating that actin and microtubule cytoskeletal networks are involved in this process. However, NEDD4, the key enzyme that regulates the Cx43 degradation pathway, is not changed, indicating that NEED4 is not involved in the upregulation of Cx43 under the stretch condition. Therefore, we conclude that there are functional relationships among Cx43, TGF beta1, actin, and microtubule cytoskeletal networks under the stretch condition.
